# Research methodology education in Europe: a multi-country, cross-disciplinary survey of current practices and perspectives

**DOI:** 10.1186/s41073-025-00183-x

**Published:** 2025-11-17

**Authors:** Silke Kniffert, Ivan Buljan, Flavio Azevedo, Peter Babinčák, Lucija Batinović, Thomas Rhys Evans, Sara Garofalo, Christopher Graham, Lucianne Groenink, Malika Ihle, Miloslav Klugar, Lucia Kočišová, Michal Kohút, Nikolaos Kostomitsopoulos, Seán Lacey, Anita Lunić, Ana Marušić, Thomas Nordström, Charlotte R. Pennington, Daniel Pizzolato, Ulf Toelch, Marta Topor, Miro Vuković, Michiel R. de Boer

**Affiliations:** 1 Berlin Institute of Health at Charité- Universitätsmedizin Berlin, QUEST Center for Responsible Research, Charitéplatz 1, 10117 Berlin, Germany; 2 Department of Psychology, Faculty of Humanities and Social Sciences, University of Split, Poljička cesta 35, Split, Croatia; 3 Department of Primary and Long-term Care, University Medical Center Groningen, Groningen, the Netherlands; 4 Department of Interdisciplinary Social Sciences, University of Utrecht, Utrecht, the Netherlands; 5 Institute of Psychology, Faculty of Arts, University of Presov, Presov, Slovakia; 6 Department of Behavioural Sciences and Learning, Linköping University Sweden, Linköping, Sweden; 7 School of Human Sciences and Institute for Lifecourse Development, University of Greenwich, London, UK; 8 Department of Psychology, University of Bologna, Bologna, Italy; 9 Royal College of Physicians of Edinburgh, Edinburgh, Scotland; 10 Division of Pharmacology, Utrecht Institute for Pharmaceutical Sciences, Utrecht University, Utrecht, the Netherlands; 11 LMU Open Science Center, LMU Munich, Munich, Germany; 12 Cochrane Czech Republic, Czech Republic: A JBI Centre of Excellence, Czech GRADE Network, Institute of Health Information and Statistics of the Czech Republic, Prague, Czech Republic; 13 Center of Evidence-based Education and Arts Therapies: A JBI Affiliated Group, Faculty of Education, Palacký University Olomouc, Olomouc, Czech Republic; 14 Faculty of Education, University of Trnava, Trnava, Slovakia; 15 Faculty of Philosophy and Arts, Trnava University, Trnava, Slovakia; 16 Biomedical Research Foundation Academy of Athens, Athens, Greece; 17 Munster Technological University, Cork, Ireland; 18 Faculty of Humanities and Social Sciences, University of Split, Split, Croatia; 19 Center for Evidence-based Medicine, University of Split School of Medicine, Split, Croatia; 20 Department of Psychology, Linnaeus University, Vaxjo, Sweden; 21 Aston University, Birmingham, UK; 22 European Network of Research Ethics Committees (EUREC), Bonn, Germany; 23 University of Split School of Medicine, Split, Croatia

**Keywords:** Research methods, Teaching research methods, Research quality, Higher education, Teaching formats, Teaching assessments

## Abstract

**Background:**

Research methodology education aims to equip students with the foundational knowledge of robust scientific practices, emphasizing deep understanding of scientific inquiry, integrity, and critical thinking in research practice. A literature review reveals that the observed diversity in research methods course design and instruction stems from a lack of consensus about the essential foundations required to critically engage with, design, and execute research in education. This is further compounded by a limited pedagogical innovation. However, no study has yet investigated how research methodology is taught and perceived across European universities. The objective of this study is to examine practices and attitudes regarding teaching research methodology in different European countries, across different disciplines and different training stages to identify commonalities and discrepancies.

**Methods:**

A cross-sectional survey was designed based on the Structure of Observed Learning Outcome (SOLO) taxonomy and further developed in several rounds of expert input and feedback, ensuring comprehensive inclusion of diverse teaching formats and assessment types. The survey was distributed to research methodology and non-research methodology higher education teachers across Europe through stratified and snowball sampling methods.

**Results:**

The survey was completed by 559 respondents across 24 countries and seven disciplinary categories. The findings identified a predominant reliance on traditional passive teaching formats, such as face-to-face or online lectures. Active methods such as flipped classroom (8.4% Bachelor, 4.8% Master, 2.3% PhD) and protocol writing (8.2% Bachelor, 6.6% Master, 3.9% PhD) were less frequently used. Written exams dominated assessment strategies at all levels. Across our stratification levels, all topics were rated very important, with hypothesis formulation, research integrity, and study design as the most necessary topics, while pre-registration, peer review, and data management plan were prioritized slightly less.

**Conclusions:**

These findings reveal relative homogeneity in research methodology teaching across academic levels and disciplines in Europe. The persistence of passive teaching formats and the limited adoption of active methodologies reflects an untapped opportunity to improve the effectiveness of research methodology education in fostering critical thinking and ethical practices. Higher education institutions need to reevaluate research methodology curricula to better align with contemporary research demands.

## Background

The quality and reliability of scientific research is subject of many international debates, with widespread concerns about the failure to replicate key findings across various disciplines. These concerns, often referred to as the “reproducibility crisis”, have been linked, in part, to methodological weaknesses, inadequate statistical practices, and insufficient transparency in research processes [1, 2]. In response, there is growing consensus that education and training in research methodology (RM) as the systematic rationale underlying a research project, integrating epistemological assumptions, conceptual frameworks, and the strategic alignment of research objectives constitute one of the most effective and necessary responses to this crisis. This is distinct from research methods, which pertain to the concrete practices employed in the collection and analysis of data [3].

RM education in Higher Education Institutions (HEIs) plays a pivotal role in shaping the future of academic research and societal progress [4]. By fostering high-quality, ethical, and innovative research practices, RM education equips students with critical thinking skills and evidence-based decision-making capabilities essential for addressing complex global challenges [5–7]. This educational foundation not only may mitigate research misconduct and promote reproducibility but also cultivates a new generation of responsible data users and analysts.

As such, HEIs should prioritize comprehensive coverage of core RM topics, provide practical training opportunities, implement meaningful assessment methods, and ensure instruction by skilled educators [5–8].

From a holistic and pedagogical perspective, RM education is expected to vary across academic levels, with an increasing degree of complexity and engagement [9]. At the Bachelor level, RM education typically employs more passive teaching formats, introducing fundamental concepts. As students’ progress to Master and PhD programs, the pedagogical approach is expected to shift towards more active, student-centered learning, where students are encouraged to shape their learning process through active participation, emphasizing critical analysis and independent research skills. Active learning strategies as well as hybrid approaches combining traditional and active formats have been shown to enhance student engagement, and motivation in order to achieve transfer of knowledge into professional practice [10–16]. This progression aligns with the goal of preparing students to conduct autonomous research. When students advance, open research practices, as an integral component of RM education, are needed to establish a “foundation for a more reproducible and inclusive science (Azevedo, 2022, p. 2)”, enhances research quality, and promotes equity [17, 18].

Since the establishment of the European Higher Education Area (EHEA) [19] in 2010, one might anticipate a harmonized approach to RM education across European countries. However, a large cross-national survey for monitoring educational policies, practices and political reforms in general revealed substantial variability across Europe [20]. Results indicate that European countries often take different paths in addressing educational challenges. Another study on higher education examined the tensions between curriculum regulation and deregulation across several European countries [21]. A comparative analysis of different national approaches to curriculum flexibility and autonomy showed an association of national differences with countries’ decentralization extent. For example, Finland’s flexible core curriculum model contrasts sharply with France’s centralized science curriculum reforms. Looking at RM education in particular, the wide range of options in designing and teaching RM is indicated by a systematic review by Matos and colleagues who point out that “*… the apparent variety reflects a lack of clarity…(Matos, 2023, p.20)” *[22]. We use the term heterogeneity to refer specifically to descriptive variation in RM teaching, rather than to normative or conceptual differences. Heterogeneity across disciplines and academic levels is both necessary and appropriate, as it reflects the distinct epistemological frameworks and methodological conventions inherent to each field of study. Variation can also be anticipated as part of the differentiation processes among HEIs, particularly in response to increasing competitive pressure within the sector. Nonetheless, within each discipline and at each academic level, internal coherence in teaching practices, RM topics, and assessment formats is essential. Such coherence should be guided by clearly articulated learning outcomes that progressively advance in complexity from the Bachelor to the Doctoral level. This approach reflects Biggs and Tang’s (2011) model of constructive alignment, which integrates learning outcomes, teaching methods, and assessment to ensure pedagogical coherence and effectiveness harmonized across European borders [9, 23]. Institutions that underscore the significance of collaborative, student-centered approaches may facilitate more consistent educational outcomes, particularly when such methods are systematically integrated into teaching and learning practices [24]. However, there is currently a lack of evidence regarding the extent to which these approaches are employed specifically in the teaching of RM. To enhance the effectiveness of RM education across European institutions, systematic discussion and evaluation of teaching practices is needed [25]. A better understanding of the scope and type of consistencies and inconsistencies in the RM practice could help identify priority targets for change and give us a sense of what 'new' or contemporary practices and content has been adopted.

To summarize, RM education within HEIs is pivotal in ensuring that early-career researchers possess the requisite skills for conducting rigorous research thereby contributing to advancements in their respective fields and enhancing research quality. This can be achieved through a pedagogical shift whereby instructional practices evolve from more passive, teacher-centered approaches in the Bachelor level towards increasingly active, student-centered learning as students’ progress to higher academic levels. The curriculum should evolve accordingly, beginning with foundational RM content and progressively incorporating more advanced topics, including open science practices and research data management. However, the extent to which this pedagogical and curricular progression has been implemented across European HEIs remains insufficiently explored. Despite widespread declarations, initiatives, and the recognized importance of RM education, there is a notable lack of clarity regarding the current state of RM teaching and standardized guidance of RM education in Europe that define core principles. Specifically, there lacks a systematic and comprehensive analysis of RM curricula, variations across academic levels, and the pedagogical practices and assessment methods employed in RM education.

Our cross-sectional survey on RM education aims to address this knowledge gap, through the following five research questions:

1. What is the estimated student workload related to RM education at different academic levels: (Bachelor, Master, PhD)?
2. Which topics are perceived to be important for inclusion within RM education and to what extent do these differ between teachers of RM and teachers of other subjects, and between geographic regions and disciplines?
3. Which teaching practices are used in RM education at different academic levels and how do geographic regions and disciplines compare?
4. Which types of course assessment are used in teaching RM at different academic levels and how do geographic regions and disciplines compare?
5. How do teachers perceive the importance of RM education to students and to institutional management within different academic levels and how do geographic regions and disciplines compare?

## Methodology

This study originated from the special interest group (SIG) on research methodology education (SIG on RM education) from the Network for Education and Research Quality (NERQ) [26, 27]. NERQ serves as a platform for educators and researchers to exchange best practices, develop training materials, and promote interdisciplinary collaboration, aiming to foster responsibility in research through the transformation of education and research systems [28]. NERQ has established multiple SIGs, each focusing on specific aspects of education aimed at strengthening research quality, including the SIG on RM education. This SIG is a community working on a voluntary basis comprising 25 interested educators across Europe, with different professional backgrounds involved in RM teaching.

For this study, we designed and administered a cross-sectional survey to teachers in Higher Education in Europe, both those specializing in RM and those who teach other disciplinary areas. We aimed to include teachers across all academic disciplines.

The study was pre-registered in October 2023 on the Open Science Framework (OSF) [29].

Ethical approval (IRB approval number: 029-06/24-03/00002) for the study was granted by the Faculty of Humanities and Social Sciences, University of Split (reference number: 2181-190-24-00009).

### Theoretical framework

**Fig. 1:**
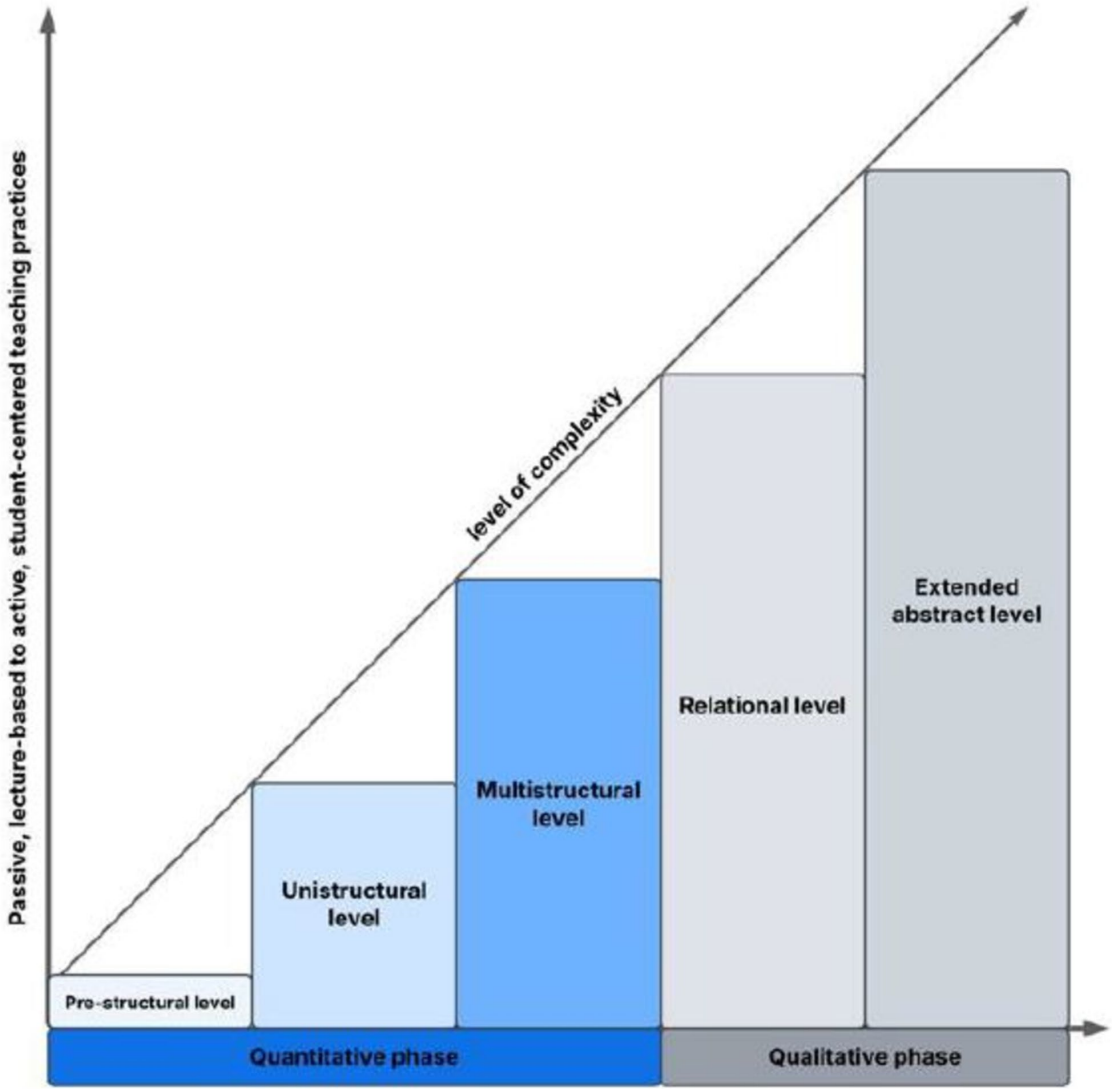
Overview of the SOLO taxonomy, adapted from Biggs and Tang [9], with the theoretical background on the x-axis and survey implementation on the y-axis

The ‘Structure of Observed Learning Outcome’ (SOLO) taxonomy was the theoretical framework used to guide the content development of the survey [9]. The concept is based on different hierarchical levels of understanding of learning outcomes that increase in complexity (Fig. 1): SOLO distinguishes between a first quantitative and a subsequent qualitative phase in learning, where the quantitative phase is aimed at increasing knowledge and the qualitative phase at deepening understanding. The quantitative phase is further classified into pre-structural, unistructural (e.g., learning terminology) and multistructural (e.g., memorizing detailed knowledge) levels where students build an initial understanding and learn discipline-dependent facts. The qualitative phase is classified into relational (e.g., explaining the relation between two or more concepts) and extended abstract (e.g., theorizing based on what is known) levels. In the last level, students exhibit the ability to apply coherent knowledge to new scenarios, interrelate different parts of their knowledge, and develop a generalized understanding.

The framework guided the comprehensive inclusion of different teaching formats and assessment types. Specifically, we included practices that ranged from those primarily aimed to enhance knowledge acquisition at a quantitative level, such as lectures, to those that focus on fostering a deeper understanding at a qualitative level, such as writing research reports or conducting peer review (Fig. 1).

To ensure comparability across academic levels and disciplines, the survey focused exclusively on classroom-based (RM) instruction. Teaching activities embedded within doctoral thesis supervision were excluded.

### Survey development and validation

An initial survey was developed by the core project team, consisting of SK, IB and MRB. This was based on the SOLO taxonomy, their knowledge of the literature and their own teaching experience. Next, subject matter experts were selected from the core project team’s networks from different disciplines. Six RM and teaching experts from the Medical Sciences, Physics, Epidemiology, Psychology and Pharmacology, and from different countries: the Netherlands, Croatia, Germany, and Brazil, critically reviewed the survey based on its content, comprehensibility, completeness, duration, length, presentation and approach.

In the third phase, the adapted survey’s content was discussed by a group of four researchers and teachers, all experts in the field and members of the SIG on RM education of NERQ. In order to reduce ethical or contextual biases, country-specific differences in the education systems, including the teaching methods and their assessment systems, were discussed and integrated into the survey.

The final phase of survey creation consisted of a pilot study among experts in higher education teaching. Nineteen experts from the project team’s networks were asked to review the survey on comprehensibility, completeness, time to complete the survey, length, presentation and practical feasibility. Of these 19 experts, we received a comprehensive review from 10 with backgrounds in the Social Sciences, Clinical Medicine, Public Health, Educational Psychology, Biochemistry, Preclinical Research, Molecular Biology, and Philosophy. These experts came from Austria, Belgium, Croatia, Czech Republic, Germany, Hungary, Sweden, and Switzerland. Based on the pilot and a final discussion session within the SIG on RM education, final adjustments were made. All reviewer feedback, on which the adjustments are based on, can be found on the OSF [30].

### Final survey content

A PDF of the final survey can be found on the OSF [31, 32].

The survey complied with data protection regulations. We obtained informed consent, alongside collecting demographic data such as gender, age group, educational degree and academic position, experience of teaching (duration), and discipline. To ensure conceptual clarity, the term *research methodology* was defined based on authoritative sources [33–35]. It then examined participants' perceptions and understanding of RM teaching, including the relevance of various RM practices, followed by a query about teaching experience across academic levels, capturing details on duration, formats, and assessment methods. Finally, the study explored educators' perspectives on the perceived importance of RM teaching to students and to the management of their institution.

### Dissemination strategy

Data collection took place from February 2024 to September 2024. In deviation from the pre-registration, we extended the data collection phase by five months, as the response rate was low due to the institutional summer break and logistical problems, such as delays in distribution.

Eligible participants included all educators affiliated with HEIs, irrespective of their educational qualifications, employment status (full-time or part-time, permanent and temporary contract), the academic levels they instructed, or their involvement in teaching RM.

**Fig. 2:**
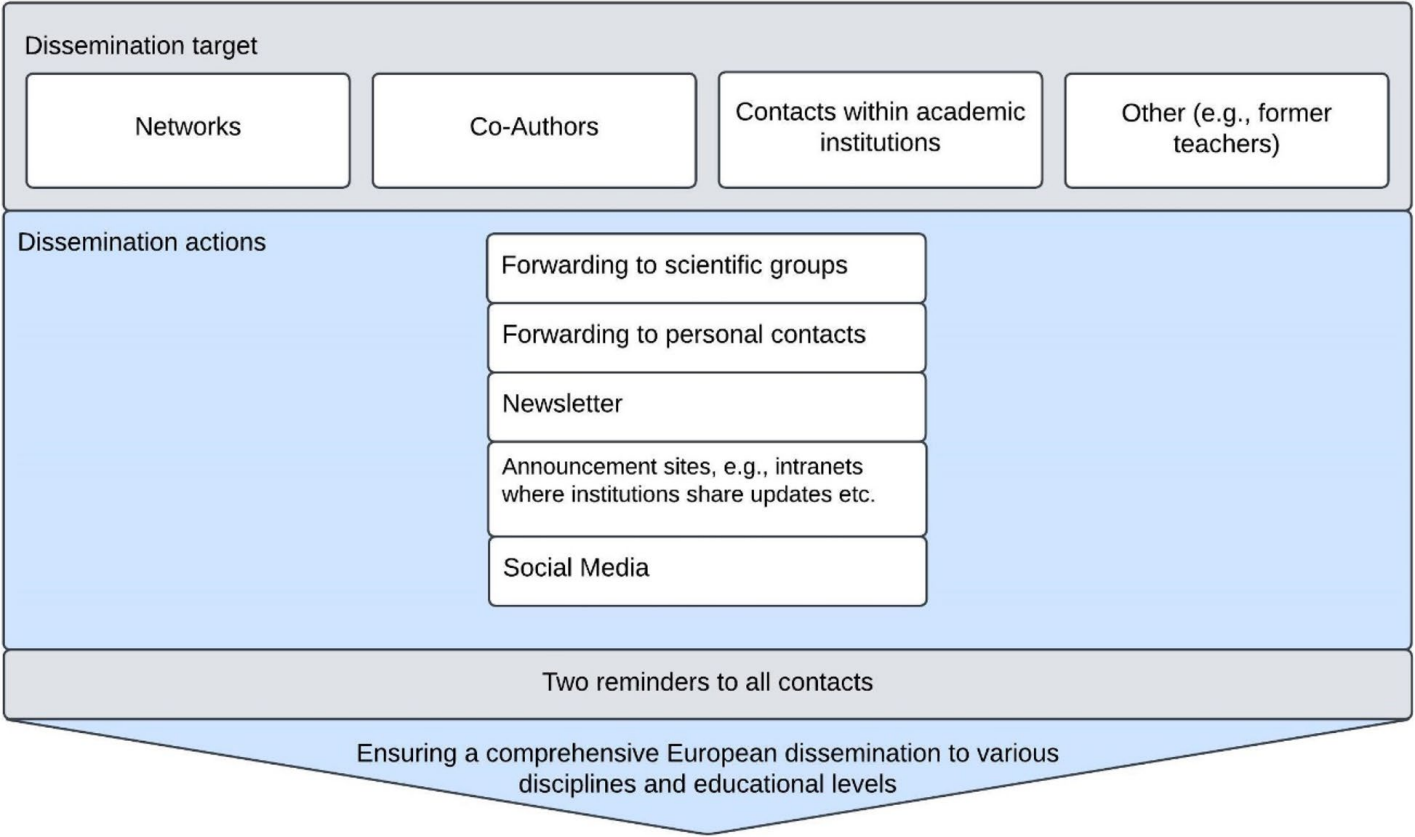
Dissemination strategy of the survey. Over a seven-month period, diverse stakeholder groups (dissemination target) were engaged in the distribution strategy. Dissemination utilized multiple channels, including social media platforms, organized group and network activities, and individual private contacts (dissemination actions). The survey was disseminated to the same recipients on three separate occasions- first dissemination and two reminders (indicated in gray). Created in Lucid (lucid.co)

We used a combination of stratified and snowball sampling methods (Fig. 2). Teachers known to the authors were contacted by email with a request for dissemination. Additional participants were recruited indirectly through professional networks and institutional channels. Initially, established networks such as NERQ, with which the project team maintained active collaborations, were engaged to facilitate dissemination. The survey was further circulated through the expert groups involved in its validation, as well as the professional networks of the co-authors, targeting diverse representation across disciplines, geographic regions, and educational levels. Later national Reproducibility Networks (RNs) of Europe were involved. Snowball sampling was used to extend the outreach.

During the seven-month data collection period, two reminders were issued to encourage distribution. Co-authors and other stakeholders, who were asked to distribute the survey in their networks, had the option to translate the survey. Only one working group translated the survey but did not provide a back translation.

### Data processing

In light of the final dataset and to enhance conceptual precision, a series of targeted methodological adaptations and data manipulations were implemented to comply with the pre-registered protocol and to avoid bias introduction in subsequent analyses.

The first adaptation involved a minor but well-justified refinement of the first research question, constituting a slight deviation from the preregistered protocol. The initial question “What is the estimated student workload for teaching RM at different academic levels (Bachelor, Master, PhD)?” was revised, based on reviewer feedback, to clarify that the students were not acting as teachers themselves. The revised question reads: “What is the estimated student workload related to RM education at different academic levels (Bachelor, Master, PhD)?”

**Fig. 3:**
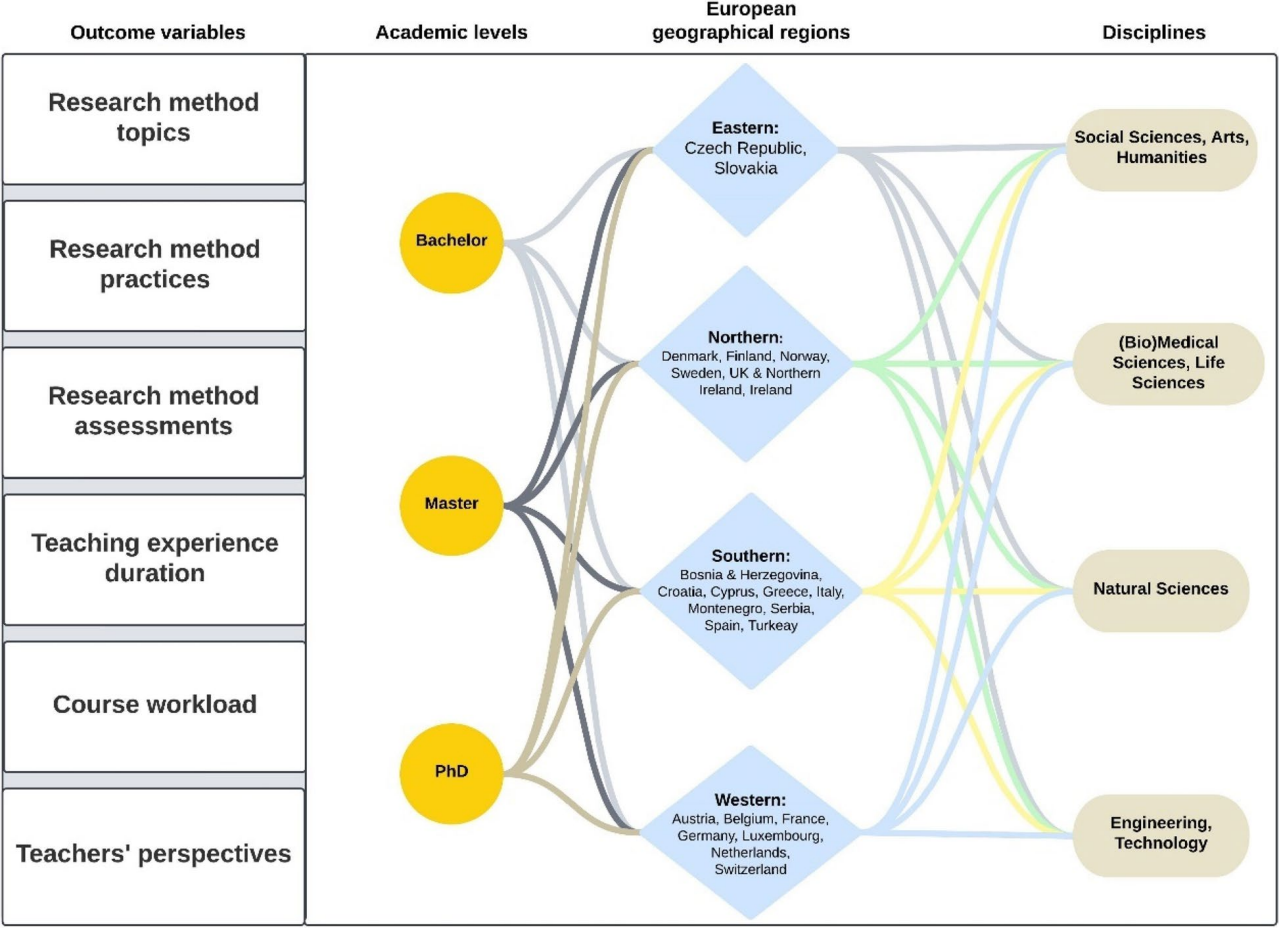
A structural overview of the study outcomes (left) with their different levels of stratification (from left to right): academic level (Bachelor, Master and PhD); four European regions (divided into Eastern, Northern, Southern and Western Europe); and the disciplines—with which RM teaching variables are analyzed. A PDF of the final survey can be found on the OSF [32]. Created in Lucid (lucid.co)

The pre-registration objectives of including at least 5% of the sample from each stratification level could not be achieved due to an overall lower response rate than expected and aimed for. We therefore aggregated countries into Eastern, Northern, Southern and Western Europe on the basis of the United Nations Geoscheme [36] (Fig. 3).

The survey was translated by one stakeholder group, which may have contributed to the highest response rate from a country in our study. However, in the translation process, the fields of Social Sciences, Arts, and Humanities were aggregated into a single category. As a result, we could not adhere to the European Union's 'Field of Science and Technology' classification, as promulgated by the 'Interoperable Europe' initiative of the European Commission [37, 38]. We therefore aggregated the three disciplines systematically across the entire dataset (Fig. 3).

To facilitate a more distinctive interpretation of the results pertaining to teaching formats and to elucidate clearer conclusions, we also categorized our *a priori* defined teaching formats (see Table S1). Specifically, we dichotomized these formats into two categories: active and passive modalities (see Table S2). The categorization was based on a contemporary definition, the methodology of which is described in detail in the supplementary materials Table S2 [39].

### Statistical analysis

The dataset was analyzed using R [20], version 4.4.0/ 2024-04-24 [40–43]. The demographic and course load data were represented in tables using frequencies and percentages. All areas of investigation were differentiated into the three academic levels of Bachelor, Master and PhD. Stratification into geographical regions and disciplines was carried out for teaching practices, course assessments and RM topics. In addition, the RM topics were stratified into RM teachers and non-RM teachers. To ensure a balanced country representation, we planned to collect at least 100 responses per country and described the ranking of topics by means and standard deviations (SD). Mean scores of the RM topics were presented including their 95% confidence intervals. The teaching practices and perspectives were presented using percentages. The course assessments and the teaching experiences were represented with medians and their interquartile ranges (IQRs). The risk of careless responding associated with social media distribution could be ruled out through outlier detection, which revealed no identifiable outlier, effectively ruling out this concern.

The dataset, the analysis code and script used to compile the results are openly available on the OSF [44].

## Results

### Demographic data

In total there were 559 teacher responses with 307 (54.9%) RM teachers, 166 (29.7%) non-RM teachers, and the remaining 86 (15.4%) respondents did not answer this question. Table 1 shows the sample characteristics.

**Table 1:** Demographic data for the entire sample, and for RM teachers and non-RM teachers separately

Variable	Level	*N*	%	*N*	%	*N*	%
Gender		Overall sample (*N*=559)		Non-RM teachers (*N*=166)^a^		RM teachers (*N*=307)	
	Female	322	57.6	100	60.2	175	57.0
	Male	227	40.6	63	38.0	129	42.0
	Do not wish to state	6	1.1	2	1.2	2	0.7
	Other	4	0.7	1	0.6	1	0.3
Age							
	21-30	39	7.0	16	9.6	16	5.2
	31-40	151	27.0	39	23.5	86	28.0
	41-50	187	33.5	60	36.1	101	32.9
	51-60	118	21.1	33	20.0	67	21.8
	61-70	53	9.5	16	9.6	31	10.1
	71-80	11	2.0	2	1.2	6	2.0
Years of teaching experience in higher education (Median, IQR^c^)		15 (8-23)		13 (13-21)		15 (9-24)	
Highest educational level achieved							
	PhD/doctorate	451	80.7	128	77.1	260	84.7
	Master/graduate or equivalent	85	15.2	32	19.3	36	11.7
	Bachelor/undergraduate or equivalent	3	0.5	0	0	1	0.3
	Other (please specify)	20	3.6	6	3.6	10	3.3
Position							
	Teaching associate (PhD student, postdoc, research fellow, research associate) with non-permanent position	102	18.3	33	19.9	48	15.6
	Adjunct teacher/visiting professor, not permanent position	15	2.7	4	2.4	7	2.3
	Assistant professor (permanent position)	135	24.2	46	27.7	66	21.5
	Associate professor (permanent position)	157	28.1	46	27.7	95	30.9
	Full professor (permanent position)	81	14.5	14	8.4	54	17.6
	Other (please specify)	69	12.3	23	13.9	37	12.1
Principal discipline^b^							
	Social Sciences, Arts & Humanities	280	50.1	79	47.6	161	52.4
	(Bio)medical Sciences & Life Sciences	157	28.1	33	19.9	104	33.9
	Natural Sciences	65	11.1	28	16.9	25	8.1
	Engineering and Technology	43	7.7	21	12.7	11	3.6
Region							
	Eastern Europe	185	33.1	72	43.4	77	25.1
	Northern Europe	96	17.2	16	9.6	71	23.1
	Southern Europe	116	20.8	37	22.3	64	20.8
	Western Europe	162	29.0	41	24.7	95	30.9

^a^86 respondents did not answer the question

^b^5 missing in non-RM teacher group and six missing from RM teacher

^c^IQR= Interquartile range

The sample comprised 322 (57.6%) female and 227 (40.6%) male participants with most of the respondents (33.5%) in the age group of 41-50 years. The majority of respondents (451/ 80.7%) had a doctorate/PhD degree, and most were Associate (157/ 28.1%) or Assistant Professor (135/ 24.2%) in a permanent position. Half of the respondents (50.1%) were from the Social Sciences, Arts, and Humanities. The largest proportion of respondents (185/ 33.1%) were from Eastern Europe. RM teachers had longer teaching experience, had somewhat more senior roles and were more often from the (Bio)medical Sciences and Life Sciences than non-RM teachers (Table 1).

### Course load for teaching RM at the different academic levels

Respondents were asked to indicate their RM teaching experience in years and the students’ study hours for one RM course. The survey comprised three sections based on academic level. To account for the fact that teachers often teach across multiple educational levels, the course load questions was repeated within each section to ensure context-specific responses. The questions were optional, resulting in non-responses from over 100 RM teachers for certain items. All teachers teach or ever taught RM at Bachelor level (317/ 100%). The discrepancy between the RM sample sizes in the demographic and course load datasets stems from underreporting: Ten respondents who initially reported no experience in teaching RM, indicated RM involvement in the academic-specific question. These responses were included in the analysis, as the more specific context in which the latter responses were given is likely to have facilitated more accurate recall. We therefore consider these affirmative responses to be valid representations of actual teaching involvement. The data show that with higher academic level, there were relatively fewer RM teachers, ranging from 72.0% (228) at Bachelor level to 37.1% (92) at PhD level. A median teaching experience of nine, eight and 10 years were found for RM teachers at Bachelor, Master and PhD level, respectively (Table 2). Similarly, the data show that the median reported students’ study hours dedicated to one RM course are lower at the PhD level (24 hours) compared to the Bachelor (35) and Master (40) level. At all levels, RM was reported to be most frequently offered in stand-alone courses (51.4% at PhD level, 44.5% at Master level and 41.9% at Bachelor level) and least frequently exclusively as integrated courses (at around 23% for all levels).

**Table 2:** Frequencies of the RM course load across Bachelor, Master, and PhD levels, for RM teachers

Variable	Level	*N* (%)	*N* (%)	*N* (%)
		Bachelor	Master	PhD
Ever taught RM (*n* = 317^b^)		317 (100.0)	263 (83.0)	248 (78.2)
RM teacher		228 (72.0)	141 (53.6)	92 (37.1)
Non-RM-teacher		88 (27.8)	122 (46.4)	156 (62.9)
Years of teaching experience (*n* = 194^b^)		194 (100.0)	117 (60.3)	73 (37.6)
Years of teaching experience (Median, IQR^a^)		9 (4–16)	8 (5–15)	10 (5–10)
Number of hours dedicated to RM in program (*n* = 182^b^)		182 (100.0)	113 (62.1)	71 (39.0)
Number of hours dedicated to RM (Median, IQR^a^)		35 (10–110)	40 (12—40)	24 (11–24)
Integration of RM course (*n* = 186^b^)		186 (100.0)	119 (64.0)	72 (38.7)
	Both standalone and integrated	64 (34.4)	39 (32.8)	18 (25.0)
	Integrated	44 (23.7)	27 (22.7)	17 (23.6)
	Stand-alone	78 (41.9)	53 (44.5)	37 (51.4)

The reported hours represent the aggregate time investment required of students for a RM course, encompassing both in-class time and self-study. An integrated course refers to a curriculum structure wherein RM content is embedded within a broader disciplinary context, incorporating RM topics alongside other subject matter within a single course framework

^a^*IQR* Interquartile range

^b^n is based on completion

The reported hours represent the aggregate time investment required of students for a RM course, encompassing both in-class time and self-study. An integrated course refers to a curriculum structure wherein RM content is embedded within a broader disciplinary context, incorporating RM topics alongside other subject matter within a single course framework. *IQR= Interquartile range. **n is based on completion.

### Topics that should be included in RM education

Respondents were asked to indicate which topic should be included in RM lessons on a scale from 1 (*completely unnecessary*) to 5 (*completely necessary*). Overall, the data show that all RM topics were considered necessary by our respondents, with mean scores between 3.9 and 4.7 (Table 3). RM topics which were indicated as most necessary are hypothesis formulation and research aim setting, research integrity, and study design. In contrast, topics such as pre-registration and peer review were seen as the least necessary.

**Table 3:** Ranking of topics according to perceived necessity in RM teaching (*n* = 474)

	**Mean**^**a**^** (SD) entire sample**	**Mean**^**a**^ **(SD) RM teachers**	**Mean**^**a**^ **(SD) non-RM teachers**
Hypotheses formulation & research aim setting	4.7 (0.71)	4.7 (0.69)	4.6 (0.73)
Research integrity	4.6 (0.72)	4.7 (0.69)	4.6 (0.76)
Study design	4.6 (0.72)	4.7 (0.66)	4.5 (0.8)
Critical reading of the literature	4.6 (0.77)	4.6 (0.76)	4.6 (0.78)
Research reporting	4.6 (0.73)	4.6 (0.72)	4.5 (0.72)
Measurement	4.5 (0.78)	4.6 (0.74)	4.4 (0.84)
Quantitative qualitative analysis	4.5 (0.77)	4.6 (0.75)	4.4 (0.77)
Research ethics	4.5 (0.85)	4.5 (0.84)	4.4 (0.86)
Literature search	4.5 (0.84)	4.5 (0.83)	4.4 (0.86)
Biases	4.4 (0.83)	4.5 (0.79)	4.3 (0.88)
Sampling methods	4.4 (0.8)	4.4 (0.78)	4.3 (0.82)
Argumentation	4.4 (0.87)	4.3 (0.93)	4.5 (0.76)
Data management plan	4.0 (0.97)	4.1 (0.99)	4.0 (0.93)
Peer review	4.0 (1)	3.9 (1.04)	4.1 (0.92)
Pre-registration	3.9 (1)	4.0 (1.03)	3.9 (0.95)

^a^1-completely unnecessary, 5-completely necessary

The weighting of the necessity of the topics was almost equal between RM and non-RM teachers. The topics identified as least necessary were peer review and pre-registration for RM teachers, and data management plan and pre-registration for non-RM teachers (Table 3).

This pattern of findings was mostly consistent across geographical regions and disciplines (see Figs. S3, S4 and Table 3). Overall, the topics of critical reading and hypothesis formulation and research aim setting were indicated as very necessary across all geographical regions, while preregistration, peer review and data management plan were considered the least important in RM teaching (see Fig. S3). In a disciplinary stratification, the rankings did not vary from the previous analysis (see Fig. S4). Except for the Engineering discipline and the aforementioned topics of pre-registration, peer review and data management plan, all RM topics were assessed as necessary, exhibiting a mean score above 4 across regions and disciplines.

### Teaching practices

**Fig. 4:**
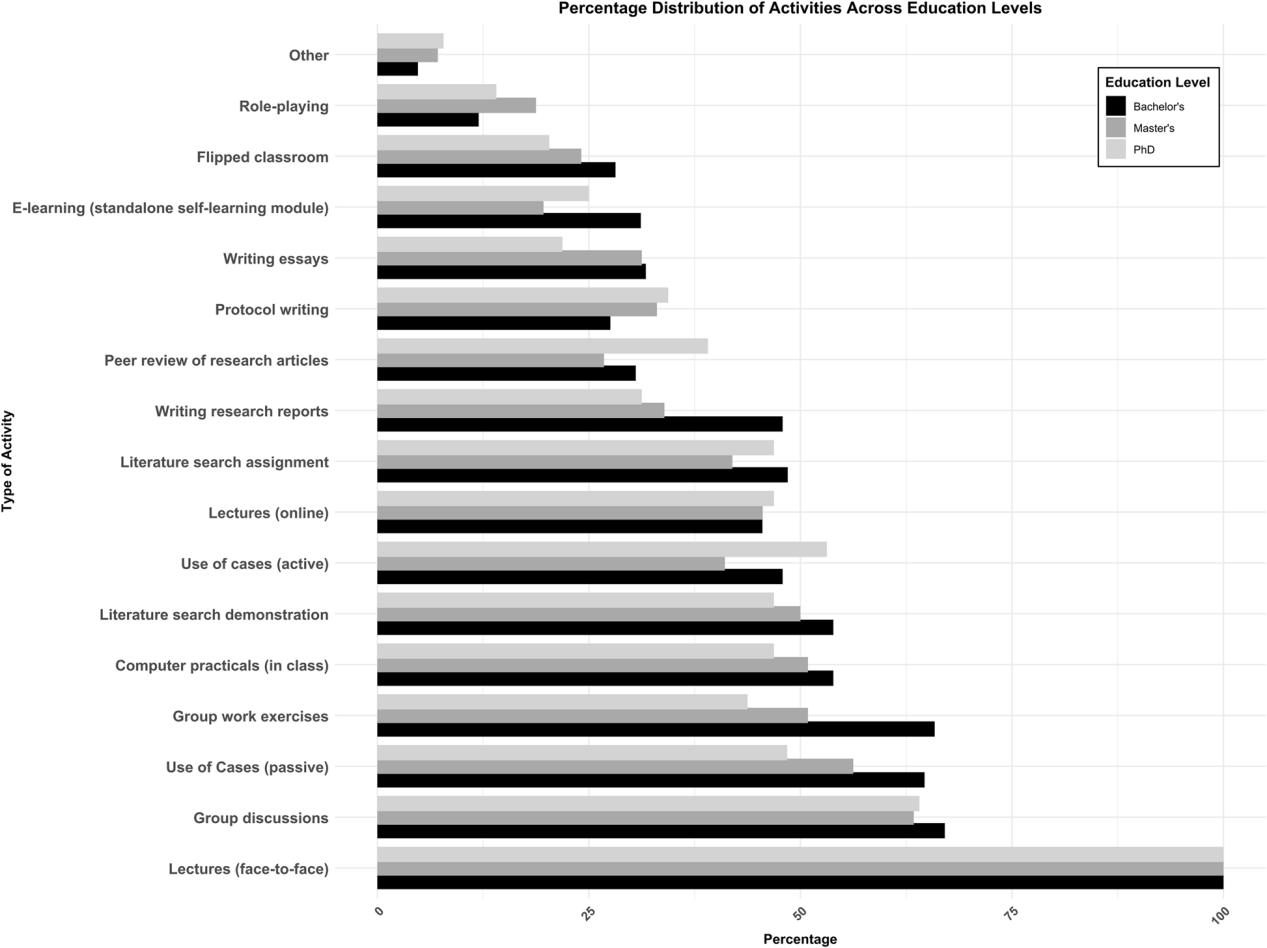
Percentages for teaching practices across educational levels: Bachelor (black, *n* = 167), Master (gray, *n* = 112), PhD (light gray, *n* = 64)

All teachers were asked to indicate all teaching practices they use in their RM courses. Overall, the most common practices were lectures, group discussions and passive use of cases (Fig. 4). Active teaching practices, such as role-playing (3.6% for Bachelor, 3.8% for Master and 1.6% for PhD level), flipped classroom (8.4%, 4.8%, 2.3%) and writing essays (9.5%, 6.3%, 2.5%) and protocols (8.2%, 6.6%, 3.9%), were much less commonly reported. There were only minor differences in the frequency of the different practices between academic levels. The definitions and categorization of the teaching practices can be found in Table S1 and S2 in the supplemental material.

The teaching practices across educational levels, stratified by region and discipline, largely mirrored the patterns observed in the overall sample (see Figs. S5 and S6). Overall, more active teaching practices were reported to be used less frequently, irrespective of geographic region or discipline.

Notable regional variations included the higher frequency of computer practicals across all academic levels in Western Europe. Additionally, in almost all European regions, lectures (online and face-to-face) were utilized relatively less frequently at Bachelor level compared to higher levels.

For higher resolution, all Figures can be found in svg format on OSF [45].

### Course assessments in the teaching of RM

All teachers were asked to indicate the relative application of course assessments they use in their RM lessons to determine the final grade. With a median of 0.0 and an IQR of 0.0- 0.0, two to four assessment methods are nearly unused at each academic level. Role playing is rarely used across all levels. Assessment methods that show greater variability and moderate central tendency are the following: Written exams with different question types and class activities were the teaching assessments with the highest median scores ranging from 5.0 to 12.5% at Bachelor and Master level. At the PhD level, students were most frequently assessed using more active methods, such as oral exams and presentations, problem-based learning assignments and literature search assignments with a median score ranging from 5 to 20%. The IQRs show wide variability in the data (see Table 4 and Fig. S7).

**Table 4:** Median percentages and IQR of forming of total grade for RM course assessments

	**Bachelor**	**Master**	**PhD**
Variables	**Median (IQR)**	**Median (IQR)**	**Median (IQR)**
Written exam (multiple choice questions)	10.0 (0.0–40.0)	10.0 (0.0–50.0)	0.0 (0.0–10.0)
Written exam (open ended questions)	0.0 (0.0–40.0)	12.5 (0.0–50.0)	0.0 (0.0–20.0)
Written exam (mixed questions)	0.0 (0.0–20.0)	0.0 (0.0–20.0)	0.0 (0.0–0.0)
Oral exam	0.0 (0.0–22.5)	0.0 (0.0–22.5)	10.0 (0.0–40.0)
Class Activity (asking/ answering questions)	5.0 (0.0–17.5)	10.0 (0.0–20.0)	0.0 (0.0–16.3)
Group discussions	0.0 (0.0–0.0)	0.0 (0.0–10.0)	0.0 (0.0–10.0)
Cases	0.0 (0.0–0.0)	0.0 (0.0–10.0)	0.0 (0.0–0.0)
Problem based learning assignment	0.0 (0.0–18.8)	1.0 (0.0–20.0)	5.0 (0.0–30.0)
Protocol writing	0.0 (0.0–0.8)	0.0 (0.0–10.0)	0.0 (0.0–12.5)
Writing essays	0.0 (0.0–30.0)	0.0 (0.0–25.0)	0.0 (0.0–20.0)
Literature search	0.0 (0.0–10.0)	0.0 (0.0–10.0)	5.0 (0.0–10.0)
Group work exercise	0.0 (0.0–10.0)	0.0 (0.0–10.0)	0.0 (0.0–0.0)
Writing research report	1.0 (0.0–34.0)	0.0 (0.0–40.0)	0.0 (0.0–23.5)
Peer review of research articles	0.0 (0.0–0.0)	0.0 (0.0–7.5)	0.0 (0.0–5.0)
Role playing	0.0 (0.0)	0.0 (0.0–0.0)	0.0 (0.0–0.0)
Computer practical assignment	0.0 (0.0–15.0)	0.0 (0.0–5.0)	0.0 (0.0–0.0)
Oral presentation	10.0 (0.0–20.0)	1.5 (0.0–20.0)	20.0 (0.0–25.0)

Bachelor, Master and PhD. For the purpose of the study, course assessment is defined as an approach to monitor if students have achieved the learning outcomes and to what extent [46]

The analysis of course assessment data, stratified by region and academic discipline, revealed that written exams (comprising various question types), writing research reports and essays were the most frequently used assessment methods across all academic levels (see Figs. S8 and S9). All participants teaching at the PhD level in Northern Europe reported the use of written exams as an assessment method. In Southern Europe, writing essays was most frequently employed at the PhD level, with a maximum median score of 60. More active forms of assessment, such as role-playing, peer review, or using cases, were either used infrequently or not implemented across educational levels. 76.5 to 82.3% of all assessments were rarely to not used at all in Eastern Europe and within the disciplines of Social Sciences, Arts, and Humanities. Consistent with the analysis of the entire sample, the data show substantial variability.

### Teaching perspectives

**Fig. 5:**
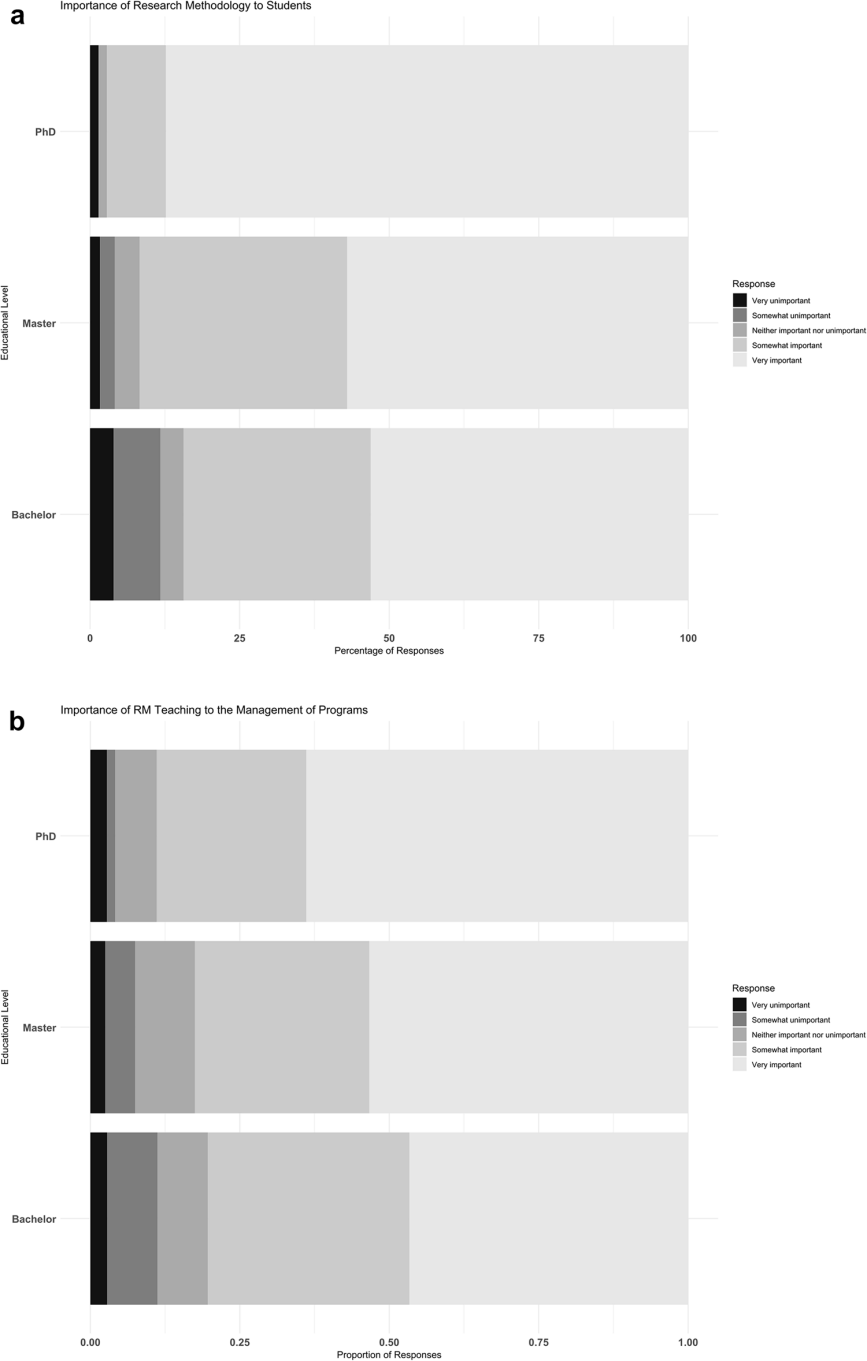
**A** Percentage of teachers with their perspectives on the importance of RM to students: (from light gray to black) Very unimportant, somewhat unimportant, neither important nor unimportant, somewhat important and very important. **B** Percentage of teachers with their perspectives on the importance of RM to the management of the institution: (from light gray to black) Very unimportant, somewhat unimportant, neither important nor unimportant, somewhat important and very important

Participants were also surveyed regarding their perspectives on the perceived importance of RM teaching to students and to the management of their institution. Overall, RM teaching was perceived as ranging from moderately (“somewhat”) important to “very important” for students (84–96%) and for institutional management (80–88%). Furthermore, Figure 5A shows that RM teaching is considered "very important" by more than 85% of teachers at the PhD level, compared to 56% at the Master level and 52% at the Bachelor level. Conversely, RM teaching was rated as "very unimportant," "somewhat unimportant," or of neutral importance by 4% of PhD teachers, 10% of Master teachers, and 16% of Bachelor teachers. Regarding institutional management, at the PhD level, RM teaching was perceived as “very important” by 63% of respondents, while at the Master and Bachelor levels, the “very important” rating was given by approximately 50% of respondents (Fig. 5B).

The perceived importance of RM teaching across educational levels, stratified by geographical region and discipline, largely mirrored the trend observed in the overall sample (see Figs. S10 to S13).

**Fig. 6:**
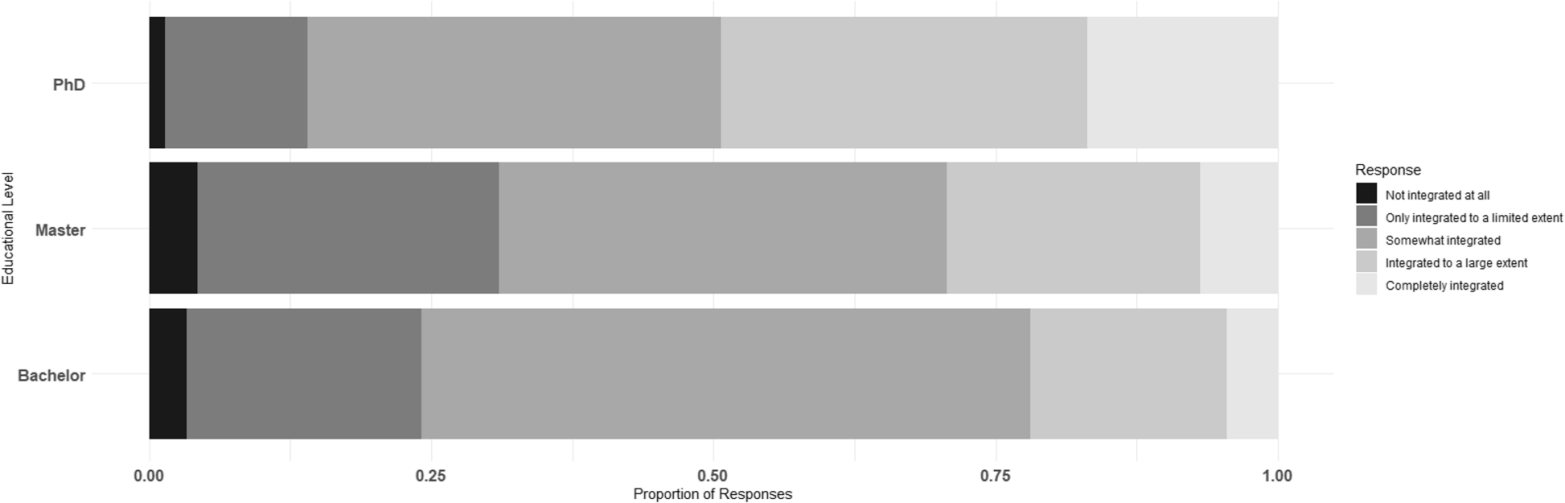
Frequency distribution of the integration of RM into other courses across Bachelor (bottom), Master (middle) and PhD level (top): (from black to light gray) not integrated at all, only integrated to a limited extent, somewhat integrated, integrated to a large extent and completely integrated

Finally, we found that the majority of respondents indicated that there was a general integration of RM education, with fewer than 10% suggesting it was not integrated at all and 10-18% suggesting it was completely integrated across all academic levels. The majority of respondents reported that RM is moderately (“somewhat”) integrated into other courses (Fig. 6).

The findings on RM integration, when stratified by region and discipline, align with the pattern of the entire sample. Across Europe, RM is most frequently reported as moderately integrated into other courses across all regions and disciplines. Comparisons between regions and disciplines revealed minor differences in the extent of integration. At PhD level, the stratified results were particularly consistent, with only negligible variations observed (see Figs. S14 and S15).

## Discussion

### In-depth exploration of the individual topics

Our survey of 559 teachers of higher education showed that most RM courses are primarily taught as stand-alone courses, with students dedicating fewer study hours per RM course at the PhD level compared to lower levels. All RM topics were generally deemed necessary, with hypothesis formulation, research integrity, and study design rated most essential, whereas pre-registration, peer review and data management planning were considered comparatively less critical across all samples and stratification levels, although still rated high. Teaching practices are dominated by lectures, group discussions, and passive use of cases; active methods such as role-play, flipped classroom, and essays and protocol writing were infrequently employed, regardless of the discipline or the academic level. We observed that the majority of assessment methods were mainly traditional, with written exams and writing research reports being the highest rated assessment forms. RM teaching was widely perceived as very important for both students and institutional management, with perceived importance increasing at higher academic levels.

Overall, the data show limited variation in the design and practical execution of teaching RM across discipline and academic levels. Education is anticipated to differ across academic levels, progressively incorporating greater complexity and fostering deeper engagement as students advance. Furthermore, RM education is expected to vary across disciplines, reflecting distinct subject-specific and research-oriented emphases tailored to the unique methodological requirements of each field. In contrast, our findings show that RM teaching in European higher education is relatively uniform and only small differences across all examined academic levels and disciplines were observed. This consistency may be largely attributed to the widespread adoption of international frameworks for research training like the European Research Area initiative [47], as well as national benchmarks such as the UK's Quality Assurance Agency guidelines [7]. These frameworks promote harmonization in curriculum design, assessment, and teaching practices to ensure comparability of skills and qualifications across institutions and countries. Additionally, the widespread adoption of guidelines, the expansion of collaborative projects, and increased public sharing of teaching materials may have contributed to reduced variability in teaching methodologies and curriculum design. These harmonization efforts are intended to ensure comparability, transparency, and quality assurance. However, our data show that the uniformity in RM teaching practices, topics, and assessments across European higher education observed exceeds such intentions. Whereas such homogeneity within individual disciplines and respective academic levels may support the alignment of methodological approaches and targeted learning outcomes, the current educational landscape inadvertently appears insufficiently responsive to the diversity required to address the distinct needs of various disciplines and academic levels, thereby limiting the potential of RM teaching as the ability to contribute meaningfully to students’ academic, professional and intellectual development.

Our findings indicate an underutilization of active teaching formats in RM education throughout European higher education, highlighting an untapped opportunity to transform our pedagogy approach to make RM teaching more engaging and relevant. Active teaching practices such as group discussions, group work exercise, and computer practicals, are moderately implemented in the aggregate sample and across stratified subgroups. Concurrently, other active practices such as role-playing or flipped classrooms remain underutilized, despite their pedagogical value [48]. A higher prevalence of active, participatory teaching approaches might be anticipated particularly at the PhD level, where advanced critical thinking, greater autonomy, and research proficiency are emphazised [49, 50]. However, this was not substantiated by our data. Lecturing as a passive or unidirectional teaching method is still one of the most widespread and most frequently practiced methods in RM teaching. It is characterized by teacher-centered instruction where students receive information, either in-person or pre-recorded, as the most passive form of teaching formats [51, 52]. The results indicate an emerging shift toward the adoption of combined teaching format concepts, wherein traditional lecture-based instruction is fully retained while being supplemented by a limited number of active teaching practices. This combined approach, supported by a few studies, highlights the potential for synergistic integration of passive and active practices to enhance academic performance [15, 16]. Nonetheless, the underrepresentation of active methods reflects a positive yet limited pedagogical development. Our findings align with findings from the European University Association's (EUA) 2018 report and the 2024 Bologna Process Implementation Report, which highlight that while there is a growing discourse on innovative teaching methods, the adoption of active methods is meagre across institutions [19, 53]. For instance, the EUA report notes that flipped classrooms were ‘fully used’ by only 15% of HEIs. These observations point to a persistent gap between policy rhetoric and institutional practice. This disconnect is particularly salient in the context of the European Higher Education Area (EHEA) [19], whose establishment has placed pedagogical models under greater scrutiny and raised concerns over the enduring predominance of lecture-based instruction in higher education [10]. Empirical evidence accumulated over the past three decades has elucidated the benefits of active learning strategies in enhancing student engagement, comprehension, and academic performance and demonstrated the limitations of exclusively traditional pedagogical approaches in facilitating autonomous work [11–14, 49, 54–57]. These findings suggest that a paradigm shift in educational methodologies is warranted to optimize learning outcomes and development in contemporary educational settings. The implementation of active learning strategies as “...a student-centered approach to the construction of knowledge focused on activities and strategies that foster higher-order thinking...(Doolittle, 2023, p.18)” is of critical importance in this context [39]. Therefore, EHEA policies emphasize the foundation of student-centered learning in “...learning outcomes-based curricula (Dakovic, 2020, p.568)” [58]. Our results did not reveal variation in teaching formats, but suggest that challenges persist in enhancing didactic approaches [53]. One explanation for the observed findings could be institutional and financial pressures that disincentivize pedagogical innovation. Universities often operate under tight budgets, making large-scale lectures more cost-effective than resource-intensive active learning approaches. Additionally, faculty promotions and research funding are often tied to publication output rather than teaching quality, reducing incentives for instructors to invest in innovative pedagogy [58, 59]. Furthermore, the implementation of active teaching formats in HEIs is often hindered by the fragmented and ambiguous distribution of stakeholder roles with unclear responsibilities and overlapping or disconnected functions. This lack of role clarity undermines both top-down and bottom-up processes that are essential for articulating and institutionalizing active teaching objectives and aligning them with broader institutional goals [58]. The absence of clear leadership and strategic direction generates uncertainty among faculty and staff concerning pedagogical priorities, thereby impeding the widespread adoption of active learning methodologies. Beyond the mere adoption of active teaching practices, ensuring their pedagogical quality is critical to realizing their full educational potential. Successful curriculum innovation and reform require well-defined institutional objectives, financial support, the active engagement of all relevant stakeholders and a cultural shift to bringing education in the focus of high-quality research [59].

Similar to the results of the teaching practices, the data show an unexpected homogeneity in preference for RM topics across regions and disciplines. We selectively included specific Open Science topics that are most closely aligned with established practices in RM education, ensuring conceptual relevance and contextual coherence within the scope of the survey. Despite the growing emphasis in scholarly research on Open Science, topics such as pre-registration, and data management plan were rated as less necessary by our respondents. This perception overlooks community-driven initiatives that have emerged in response to the replication crisis, advocating for the inclusion of these topics to improve research integrity and reproducibility [17, 60–63]. From our experience we know that in some academic programs, statistics is integrated within RM education, whereas in others it is a separate subject. The same applies to the teaching of literature reviewing, which is often categorized under RM education, but also sometimes taught as a standalone subject. Accordingly, it might be inferred that topics such as pre-registration and data management plan are generally perceived as facilitators for advancing Open Science and reproducibility and are not classified as RM education by teachers. Alternative hypotheses posit that their adoption may be limited because RM teachers perceive these topics as less critical, overly burdensome or it may be attributed to a lack of familiarity [64], considering that the norms governing these activities have been evolving rapidly.

Despite growing attention to the development of university curricula, including cross-curricular didactic frameworks and teaching practices in recent decades [65–70], a gap remains in understanding why specific RM topics are underutilized in academic instruction. Although the survey did not focus exclusively on Open Science or reproducibility, it is notable that these topics were consistently rated low. To address our findings, tentative recommendations may therefore be warranted. This trend raises concerns about whether RM education adequately prepares students to engage with transparent research practices, which are essential for mitigating issues like publication bias and *p-*hacking. Integrating these topics more explicitly into curricula could help bridge the gap between research training and contemporary scientific standards and could contribute meaningfully to enhance transparency, epistemic integrity and robustness in research. This educational shift plays a key role in elevating the standard and societal trust of scholarly research [4, 71]. Institutions could embed Open Science into RM curricula through structured modules on pre-registration, require students to engage in data management plan processes, and/or incorporate Open Science practices into thesis and dissertation projects. The alignment of RM education with active teaching formats and Open Science practices has been shown to amplify students’ educational outcomes to apply theoretical knowledge to practical research scenarios [72–75]. As described above, irrespective of the reasons for the homogeneity, the consistency we observed highlights the importance of reevaluating existing RM frameworks to align the selection of RM topics with the diverse requirements of academic disciplines and levels, ensuring that RM education remains responsive to contemporary research practices including fostering Open Science and reproducibility.

Despite the advantages of active, practice-oriented assessment methods, our results show that these approaches remain underutilized in RM education [76–79]. This underutilization mirrors patterns observed in teaching practices and RM topics, where traditional formats continue to dominate. Only approximately 20% of all examined assessment methods seem to be frequently used by RM teachers and traditional written exams remain predominantly the primary assessment method across all academic levels, irrespective of discipline or geographical region stratification. The implementation of innovative assessment strategies in higher education is often constrained by structural and institutional barriers, including limited time, insufficient support, and a lack of faculty training. Moreover, rigid policy frameworks and accreditation standards that prioritize standardized testing further impede the adoption of diverse and practice-oriented assessment approaches [80, 81]. Effectively addressing these barriers necessitates a multifaceted approach, including institutional support, revisions to accreditation standards, and a cultural shift within academia to value innovative teaching and assessment practices [80].

Respondents acknowledged the importance of RM teaching both to students and to institutional management, particularly at PhD level. However, our findings indicate that the cumulative number of hours that students spend on a single RM course is lower at the PhD-level than at Bachelor and Master level. Our data show that at the PhD level, RM teaching is perceived as very important by teachers, with over 80% emphasizing its importance to students, and more than 60% highlighting its value to institutional management. Surprisingly, however, data reveal that PhD programs allocate the least time to RM teaching, averaging 11–16 fewer aggregated hours required of students for one RM course compared to Bachelor and Master levels. This contradiction may be attributed partly to the substantially lower European Credit Transfer and Accumulation System (ECTS) requirements in PhD programs and the correspondingly reduced teaching load, which consequently results in a lower number of RM teachers compared to Bachelor and Master programs. Furthermore, this may be indicative of a presumed premise within the study programs that PhD students possess the necessary prior knowledge from earlier academic levels, enabling them to apply these competencies effectively in their forthcoming research activities. As the survey captures workload associated with a single RM course, it is important to acknowledge the potential confounding effect of students enrolled in multiple RM courses. Moreover, there is currently no empirical evidence to determine the optimal number of RM education hours required at various academic levels to foster robust research practices and outcomes in early-career researchers. Nor do we know whether these requirements should be the same across disciplines or fields of research. Our findings lead to the question whether a median study load of 24 hours students spend learning research methodology in a typical RM course, including both class time and self-study at the PhD level, is sufficient to provide PhD students with the necessary knowledge and skills at this advanced academic stage of their training and to uphold the standards of high-quality research.

The limited variation in teaching practices, and assessments across academic levels observed in this study raises important questions about how instructional approaches reflect differing levels of expected student competence. While such overlap may be due to a shared foundational core in RM education, the progression from bachelor to doctoral level typically implies increasing demands in terms of conceptual depth, analytical complexity, and independence in knowledge production. Although our study did not conduct a detailed analysis of the level of target competence and content depth, the observed consistency suggests that RM teaching may not yet be systematically aligned with the distinct learning needs at each academic stage. Previous European studies have pointed to the evolving nature of student competencies across educational levels and have emphasized the importance of developing research competencies in the different academic levels, but there remains a lack of empirical evidence specifically addressing how RM is taught in response to these changing requirements [82, 83]. Our findings provide an indication for the need for more structured and level-appropriate design of RM instruction across higher education.

### Strengths and limitations

This is the first study to survey teaching practices and perspectives on RM education in higher education across Europe, including a broad range of disciplines. As such, our results add to existing knowledge, which is primarily from studies on higher education in general or from discipline-specific studies on RM education. However, some limitations, especially related to sampling, warrant cautious interpretation of our results.

Due to the absence of an explicit screening procedure, it cannot be ruled out that individuals outside the intended target population, such as those solely engaged in supervising research projects or doctoral theses, may have participated in the survey. Nevertheless, we consider the likelihood of such occurrence to be very low and do not expect any substantial impact on the validity of our findings. The study sample primarily comprised teachers recruited through authors’ institutions, networks, and expert groups. To mitigate potential selection bias, a snowball sampling approach was employed, aiming to diversify the sample. This approach relied on the authors' contacts disseminating the survey within their networks, facilitating further distribution through multiple subsequent channels. The inability to achieve the desired response rate may have resulted in an overrepresentation of certain regions and institutions and an underrepresentation of specific disciplines such as Engineering, Technology, and the Natural Sciences, thereby introducing sampling bias and potentially distorting the findings. Aggregating countries into geographic regions was intended to enhance the exploration of regional differentiation, leveraging sample size of combined countries. This may have masked nuanced country- or discipline-specific differences in teacher attitudes and perspectives on RM teaching. The recruitment strategy may mean that participants that have an interest in RM education may have been more likely to have responded. The findings of this study reveal that passive teaching practices are used most frequently. Critical topics such as pre-registration, data management plan, and peer review are among the least necessary of RM education through all stratifications. This prompts the question of whether the findings might have indicated a different distribution of teaching practices, potentially reflecting a greater inclination towards passive and less transparent practices, had random sample recruitment been feasible. The high absolute rating of the three topics, despite being rated as the least necessary, reflects a relative hierarchy rather than an absolute lack of necessity and could be an indication of the introduction of a response style bias. Respondents may exhibit a tendency towards positive or extreme responses, which inflates the absolute scores even for lower ranked topics. Furthermore, the survey is constrained by its reliance on self-reported data, introducing uncertainty regarding the accuracy of the information such as the students’ study hours provided by respondents.

Due to the translation error, the aggregation precluded the differentiation of findings across Social Sciences, Arts, and Humanities, thereby necessitating their aggregation in the analysis of the entire dataset. This affected our ability to draw any conclusions about the underlying individual disciplines.

Furthermore, the selection of RM practices, topics and assessments was guided by expert judgment, which inherently introduced a degree of subjectivity. This may have led to the omission of certain relevant elements or the inclusion of less central ones, potentially affecting the survey’s comprehensiveness and reproducibility. While the inclusion of additional topics related to Open Science and reproducibility skills was considered by the expert panel, the decision was made to limit the scope in order to maintain participant engagement and reduce the risk of survey fatigue, while still capturing insights into the intersection of RM education and Open Science. The survey focused exclusively on formal, classroom-based (RM) teaching to ensure conceptual clarity and comparability across academic levels and disciplines. Consequently, RM-related teaching embedded in the doctoral research process, such as supervision during thesis work, lab-based mentoring, or project-specific methodological guidance, was not included in the survey. This decision was based on the recognition that such activities are often informal, highly individualized, and not uniformly classified as structured teaching. Including them could have compromised conceptual clarity and inflated estimates of RM teaching, particularly at the doctoral level, by conflating distinct forms of pedagogical engagement. At the same time, this exclusion likely led to an underrepresentation of the full spectrum of how RM is taught in practice, particularly in academic settings where doctoral education relies heavily on hands-on, research-integrated learning. By omitting the supervisory context, the survey may have missed insights into how advanced methodological competencies are developed through iterative, hands-on engagement with real-world research problems. Future research should incorporate this important dimension of RM education.

In integrated course formats, RM content is typically embedded within broader disciplinary instruction and may not be assessed through dedicated assessments. Although respondents were instructed to report on assessment methods regardless of whether the RM content was delivered in a stand-alone or integrated format, the absence of explicit RM assessment in integrated courses was not considered. This may have resulted in item non-response or ambiguity in responses, potentially introducing bias into the findings. Furthermore, this study focused on summative assessment methods to enable meaningful comparisons across different institutional, disciplinary, and national settings, but did not capture the diversity and pedagogical value of formative assessments used to support student learning. Future research should investigate formative assessment practices in RM education to build on the insights provided by this study and to develop context-sensitive understanding of how student learning is supported throughout the instructional process.

## Conclusions

In summary, our data reveal a highly uniform pattern of RM teaching in Europe, indicating minimal variation across all stratifications. Across academic levels and disciplines, there is a marked consistency in teaching practices, assessment methods, and covered topics. Notably, at the Master and PhD levels, the use of active teaching practices, assessments, and advanced RM topics beyond the fundamentals remains relatively underutilized. As such, HEIs may be squandering opportunities to cultivate students’ critical thinking, active engagement, and practical skill development. This observed all-encompassing uniformity stands in marked contrast to the harmonization sought by European Union frameworks, which aim to balance comparability with the preservation of disciplinary and institutional diversity. In such a framework, heterogeneity within RM education arises as a functional necessity, shaped by the diverse epistemological foundations and methodological approaches that characterize distinct academic disciplines, alongside the strategic differentiation pursued by HEIs in competitive contexts. In contrast, homogeneity, expressed through internal coherence at both disciplinary and institutional levels, remains essential for maintaining pedagogical consistency, methodological rigor, and standardized assessment practices. Naturally, this only refers to homogeneity in state of the art teaching and assessment formats on contemporary topics. Consequently, variation across fields fosters intellectual and institutional adaptability, while uniformity within fields upholds academic integrity and ensures replicability. This dynamic reflects the inherent balance between diversity and structure that underpins the effective functioning of academic ecosystems. Understanding the factors that led to the observed uniformity is crucial for maximizing teaching effectiveness, promoting pedagogical innovation and enhancing the quality of higher education across Europe. As previously discussed, structural constraints, such as limited institutional resources and incentive structures favoring research productivity over pedagogical advancement, should be considered in this context. Future research and policy development should focus on achieving an optimal balance between comparability and diversity, ensuring that RM education remains adaptable to the specificities of different academic levels and disciplines, but is comparable across borders (Table 5).

**Table 5:** Overview of the key outcomes derived from the data and the subsequent recommendations to improve future research culture

**Conclusions**	**Recommendations**
The low prevalence of active learning strategies suggests that RM education may not be sufficiently engaging or effective in fostering critical thinking and autonomous working	A need for pedagogical reform by encouraging active learning approaches: Universities should incorporate more participatory methods (e.g., flipped classrooms, peer review exercises)
The ratings of pre-registration and data management plan highlight a gap between RM education and contemporary research standards	Increase representation of advanced RM practices: RM curricula should integrate pre-registration, data management plan, and transparent research workflows
The uniformity of RM topics and decreasing study hours in advancing academic levels raises concerns about whether RM education adequately prepares students to engage in professional practices	Aligning curricula with students’ diverse, context-specific educational needs by selecting RM topics based on intended learning outcomes and adjusting study investment accordingly across academic levels and disciplines
There is a notable absence of established standards for RM education, including a lack of discipline-specific guidelines, evidence-based recommendations for effective assessment strategies, and clear benchmarks regarding the recommended scope and intensity of RM instruction	Development and implementation of general and discipline specific standards for the content and didactical approaches in RM education

## Data Availability

The pre-registration, the reviewer feedback, the final survey, the dataset generated and analyzed during the current study, the code and the figures in svg format are available on the OSF: https://osf.io/72xky/files/osfstorage under *CC-By Attribution 4.0 International* license.

## Abbreviations

ECTS: European Credit Transfer and Accumulation System
EHEA: European Higher Education Area
EUA: European University Association's
HEIs: Higher Education Institutions
IQR: Interquartile range
NERQ: Network for Education and Research Quality
OSF: Open Science Framework
RM: Research Methodology
RNs: Reproducibility Networks
SD: Standard deviation
SIG: Special interest group
SOLO: Structure of Observed Learning Outcome
